# Mortuary Statistics of the City of Selma

**Published:** 1874-02

**Authors:** 


We have received the "Report of the Mortuary Statistics of the city of Selma, Alabama, for a period of twenty-nine months, extending from August 1, 1871, to January 1, 1874," by Dr. Benjamin H. Riggs, Secretary of the Board of Health. The report is full and complete, and presents food for thought, for generalization and improvement, not only to the local Board of Health, but likewise to the medical profession.

Selma is represented to be in a decidedly malarial region, but Dr. Riggs asserts his belief "that Selma, for several years, can show fewer deaths, per thousand of population, than the surrounding country. There have been no epidemic tendencies at all; no prevailing types of disease; cholera and yellow fever have both been near us, but there have been no cases in the city." "I am satisfied that malaria, be it what it may, is of a heavy nature, and does not operate far from its source. Its area of influence is circumscribed by a few feet. From this belief I draw the conclusion that any city, town or country settlement, even in the malarial regions, can maintain healthfulness by attention to the laws of health, notwithstanding the disadvantages of their location."

The population of Selma is put down at eight thousand. The mortality from phthisis, forty-five; typhoid fever, thirteen; cerebro-spinal fever, thirty-five; congestive fever, nine; congestive chill, seventeen; pneumonia, twenty-one; typhoid pneumonia, four. The mean mortality for the twenty-nine months is seventeen and one-half per month. Of the forty-five who died of phthisis, fifteen were *whites*, and thirty were *blacks*. The ratio of whites to blacks, in the population, being as nine to eleven. The ratio of deaths per thousand of population is set down at four and one-sixth for the *whites*, and six and nine-elevenths for the *blacks*.

Dr. Riggs remarks: "Our climate is too humid and malarial to be a good place of residence for consumptives."

The deaths from *diphtheria* stand eleven whites to two blacks; from *cerebro-spinal fever*, (meningitis), thirteen whites and twenty-two blacks; of *old age*, seven whites to four blacks. Of the eleven, six were natives of the State of North Carolina, and the mean age of all, eighty-one and one-half years.

